# Supplementary material for: Structural identification of N-glycan isomers using logically derived sequence tandem mass spectrometry
Source: Commun Chem. 2021 Jun 17;4:92. doi: 10.1038/s42004-021-00532-z (PMC9814355; doi:10.1038/s42004-021-00532-z)
Supplement: Supplementary file 1 — Supplementary Information [file 42004_2021_532_MOESM1_ESM.pdf]

Supplementary Information

Structural identification of N-glycan isomers using logically derived sequence tandem mass spectrometry

Chia Yen Liew[1,2,3], Chu-Chun Yen[1], Jien-Lian Chen[1], Shang-Ting Tsai[1], Sujeet Pawar[4,5,6], Chung-Yi Wu*[4,5], Chi-Kung Ni*[1,3,6]

[1] Institute of Atomic and Molecular Sciences, Academia Sinica, Taipei 10617, Taiwan

[2] International Graduate Program of Molecular Science and Technology, National Taiwan University, Taipei 10617, Taiwan

[3] Molecular Science and Technology, Taiwan International Graduate Program, Academia Sinica, Taipei 10617, Taiwan.

[4] Genomics Research Center, Academia Sinica, Taipei 11529, Taiwan

[5] Chemical Biology and Molecular Biophysics, Taiwan International Graduate Program, Academia Sinica, Taipei 11529, Taiwan

[6] Department of Chemistry, National Tsing Hua University, Hsinchu, 30013, Taiwan

* Corresponding authors, e-mail addresses: cyiwu@gate.sinica.edu.tw and ckni@po.iams.sinica.edu.tw

A. Retro-Aldol Reactions

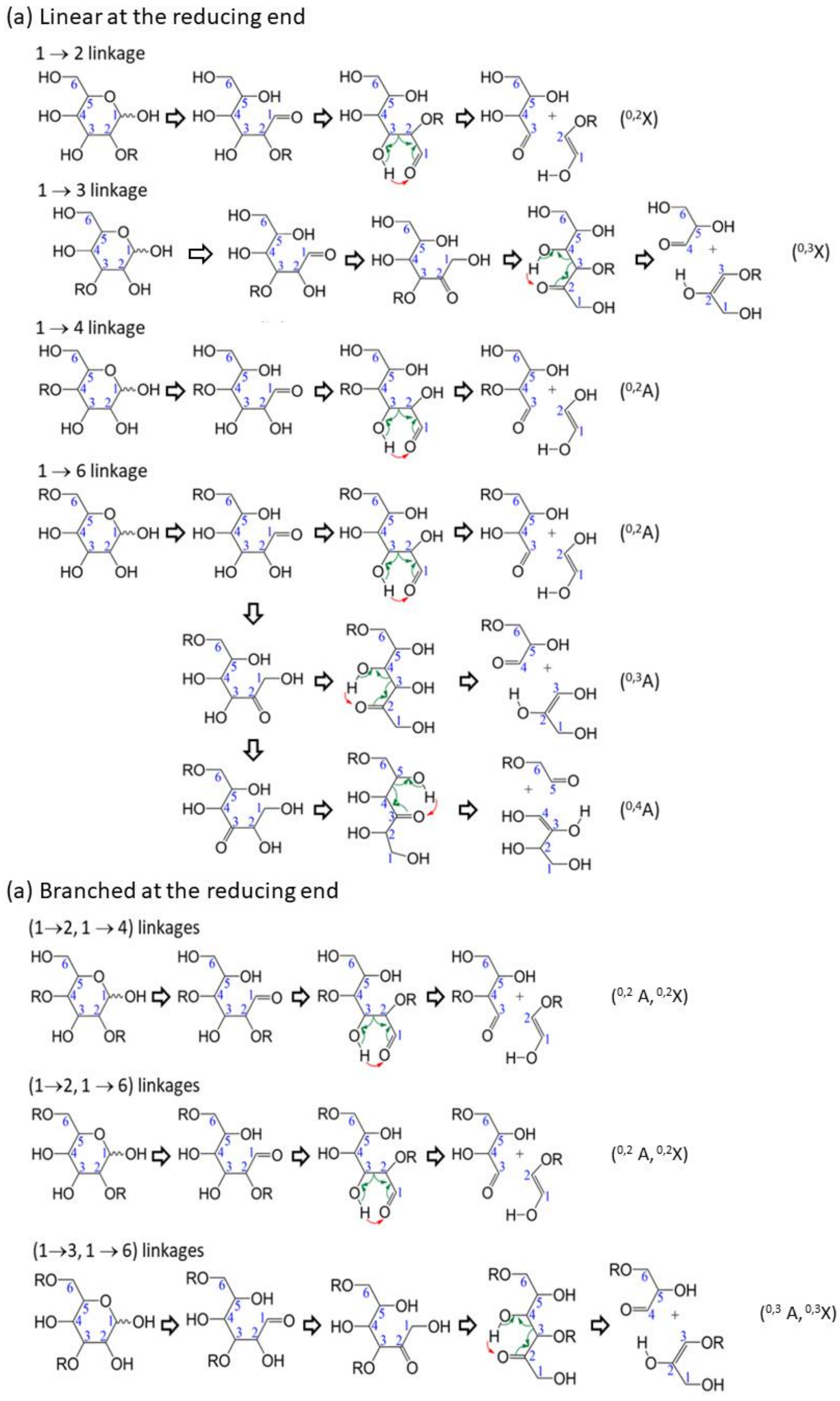

Supplementary Figure S1. Mechanism of retro-aldol reactions. Retro-aldol reactions for various linkages of hexose at the reducing end. R represents the oligosaccharide consisting of hexoses and N-acetylhexosamines.

B. CID Spectra of $Man_4GlcNAc_2$ N-glycans

$Man_4GlcNAc_2$ N-glycans have a total of seven isomers. The CID spectra of five of them are presented in the main text, and the CID spectra of the other two are displayed in Figure S2.

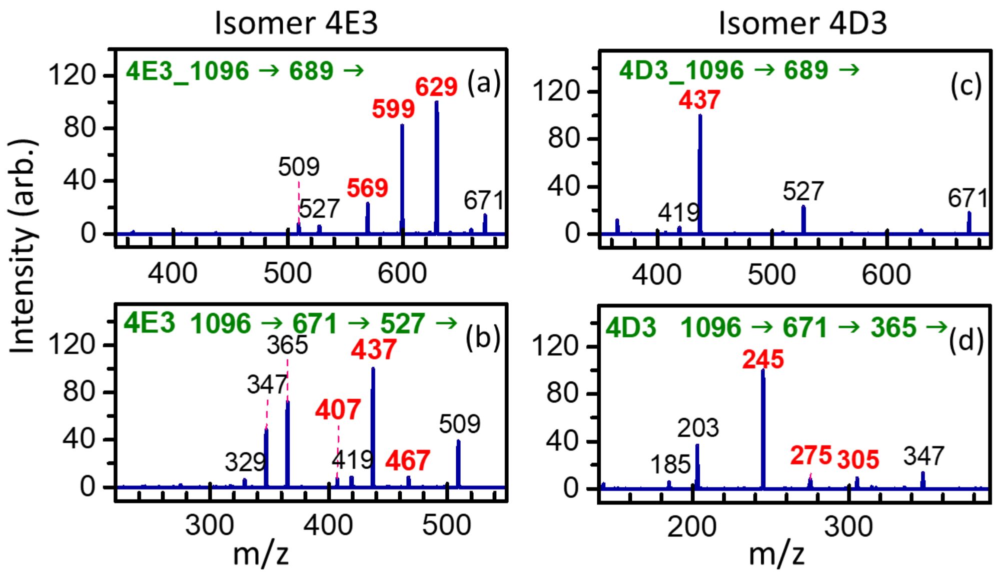

Supplementary Figure S2. CID spectra of $Man_4GlcNAc_2$ N-glycanisomers 4E3 and 4D3. (a) and (b): The intensity ratio of ions m/z 629, 599, and 569 are near the ratio of 5:3.5:1 in S2(a), indicating the isomer belongs to group E [rule 2(a)]. The intensity of ion m/z 437 is much higher than the intensities of ions m/z 407 and 467 in S2(b), indicating that the isomer is 4E3 [rule 2(c)]. (c) and (d): The intensity of ion m/z 437 is much higher than the intensities of ions m/z 569, 599, and 629 in S2(c), indicating that the isomer belongs to group D [rule 2(e)]. The intensity of ion m/z 245 is much higher than the intensities of the ions m/z 275 and 305 in S2(d), indicating that the isomer is 4D3 [rule 2(d)].

Supplementary Note 1

The α and β anomeric configurations at the reducing end of each sialylgalactose (3'-sialylgalactose or 6'-sialylgalactose) coexist in nature. We first separated these two

anomers in each sialylgalactose through HPLC and then measured the corresponding CID spectra immediately after separation. The chromatograms and the corresponding CID spectra are presented in Figure S3(a)-(f).

The assignment of anomericity to the CID spectra in Figure S3 is made through a comparison with the CID spectra of sialyllactose. The CID sequence of sialyllactose, 656→612→450, starts from the sialyllactose sodium adduct (ion m/z 656), followed by the elimination of CO_2 from sialic acid (ion m/z 612) and the generation of sodium adduct of sialylgalactose without CO_2. The CID spectra of sialylgalactose without CO_2 are illustrated in Figure S3(g) and (h). A comparison of the CID spectrum of 3'-sialyllactose in Figure S3(g) with the spectra in Figures S3(b) and S3(c) reveals that the spectrum in Figure S3(c) is similar to that in Figure S3(g), indicating that the CID spectrum in Figure S3(c) is from α-Sia-(2→3)-β-Gal and the other spectrum [Figure S3(b)] is from α-Sia-(2→3)-α-Gal. An analogous method was used to assign the anomericity to the spectra in Figures S3(e) and S3(f). Because the galactose in N-glycan was in the β configuration, only Figures S3(c) and S3(e) were used for the structural determination of N-glycans.

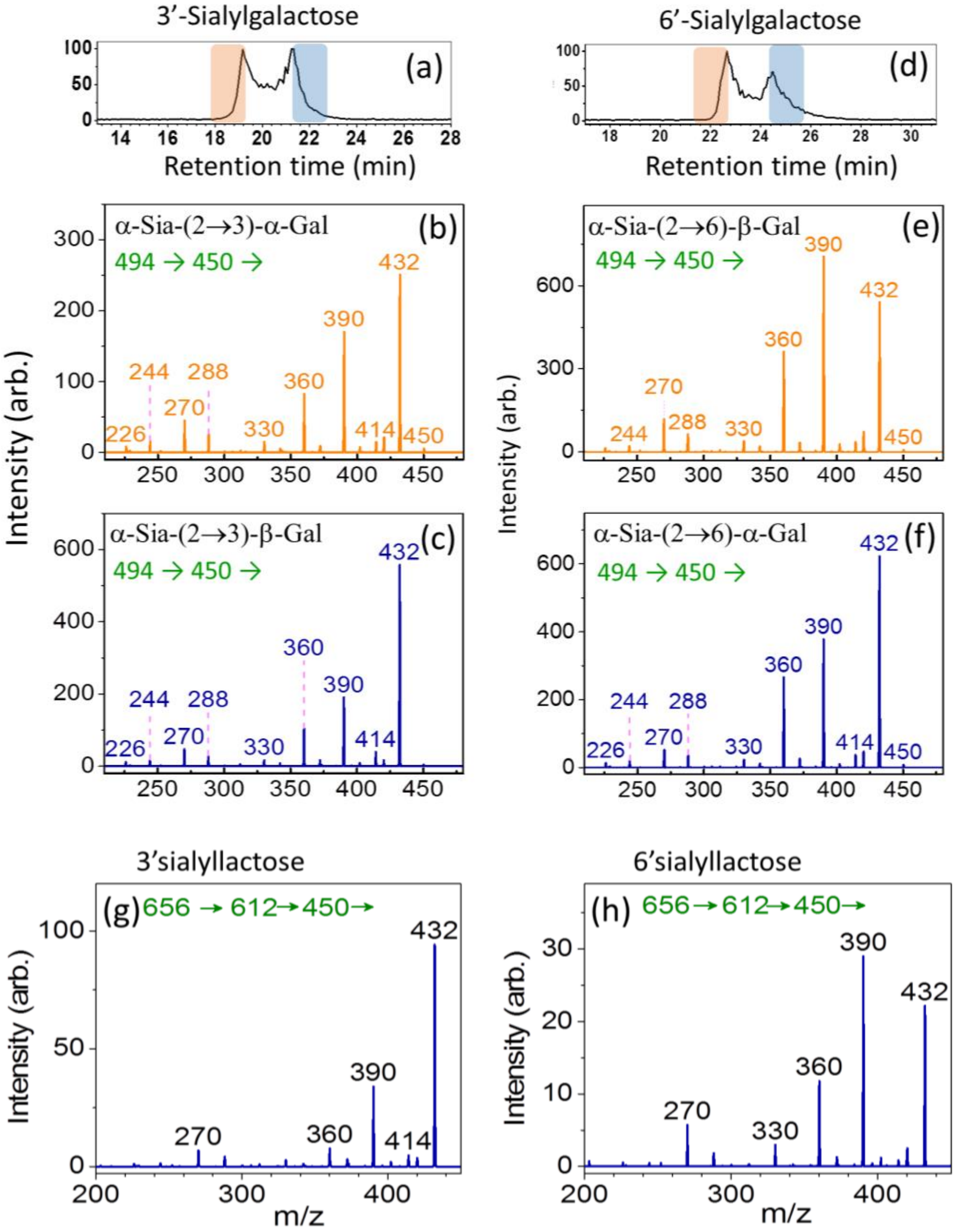

Supplementary Figure S3. Chromatograms and CID spectra of sialylgalactose and sialyllactose. (a) and (d): Chromatograms; (c)-(f): CID spectra of 3’-sialylgalactose (α-Sia-(2→3)-Gal) and 6’-sialylgalactose (α-Sia-(2→6)-Gal). The orange and blue bars surrounding each peak in the chromatograms represent the period in which the CID spectra were measured. The corresponding spectra are presented in orange and blue, respectively. (g) and (h): CID spectra of 3’-sialyllactose and 6’-sialyllactose. The CID sequence is shown in green.

C. LODES and CID of $Hex_8GlcNAc_2$ N-glycans Extracted From Soybean Proteins

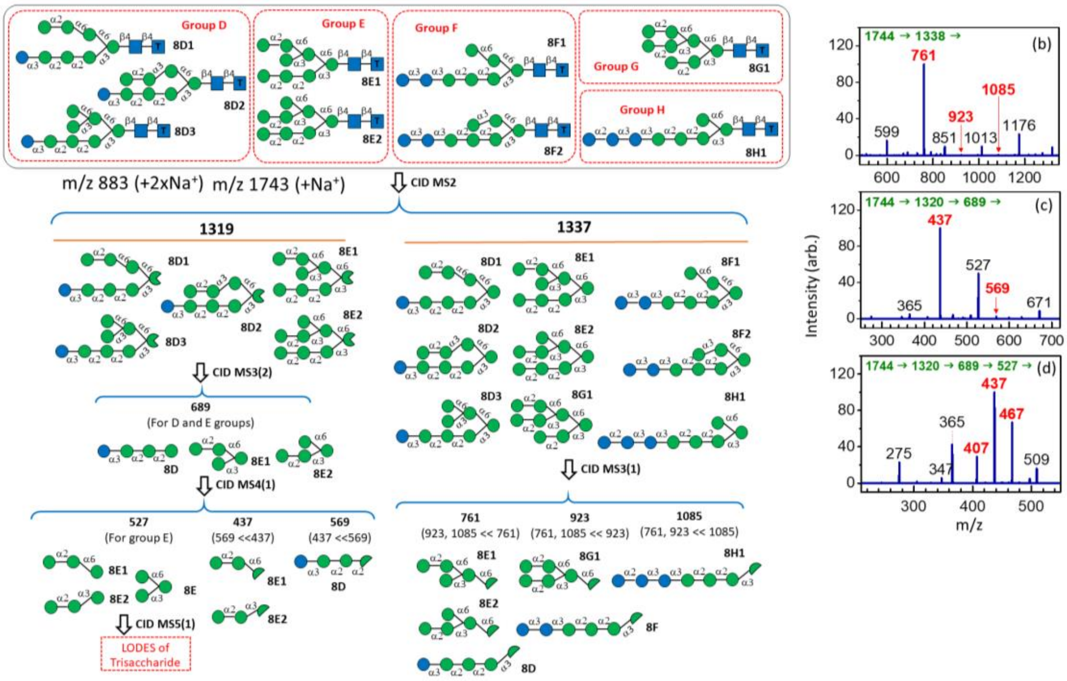

Supplementary Figure S4. LODES and CID spectra of $Hex_8GlcNAc_2$ N-glycans. (a) LODES of $Hex_8GlcNAc_2$ N-glycans. Only the LODES for the structural identification of isomer 8E1 is illustrated. (b)–(d) CID spectra of $Hex_8GlcNAc_2$ N-glycan extracted from soybean proteins. The intensity of ion m/z 761 is much higher than the intensities of ions m/z 923 and 1085 in (b), indicating that the isomer belongs to group D or E according to the LODES MS^3(1) in (a) and rule 2(e). The intensity of ion m/z 437 is much higher than the intensities of ion m/z 568 in (c), indicating that the isomer belongs to group or E according to the LODES MS^4(1) in (a) and rule 2(e). The intensity ratio of ions m/z 467, 437, and 407 is close to 4:6:1, indicating that the isomer is 8E1 according to the LODES MS^4(1) in (a) and rule 2(a).

D. CID of $Man_4GlcNAc_2$ N-glycans Extracted From Hen Egg Ovalbumin

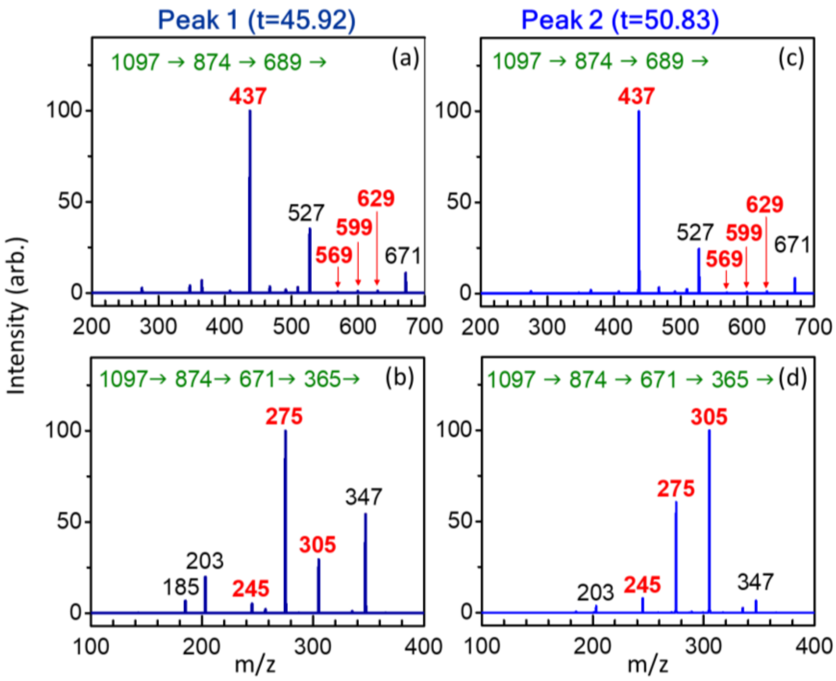

Supplementary Figure S5. CID spectra of $Man_4GlcNAc_2$ N-glycans extracted from hen egg ovalbumin. The LODES of $Man_4GlcNAc_2$ N-glycan is illustrated in Figure 1 in the main text. (a) and (b): CID of peak 1 (retention time of 45.92 min in Figure 5(b) in the main text). The intensity of ion m/z 437 is much higher than the intensities of ions m/z 629, 599, and 569 in (a), indicating that the isomer belongs to group D according to MS^3(1) in Figure 1(a). The intensity of ion m/z 275 is much higher than the intensities of ions m/z 245 and 305 in (b), indicating that the isomer is 4D2 according to MS^4(2) in Figure 1(a). (c) and (d): CID of peak 2 [retention time of 50.83 min in Figure 5(b) in the main text]. The intensity of ion m/z 437 is much higher than the intensities of ions m/z 629, 599, and 569 in (c), indicating that the isomer belongs to group D according to MS^3(1) in Figure 1(a). The intensity ratio of ions m/z 305, 275, and 245 in (d) is close to 5:3.5:1, indicating that the isomer is 4D1 according to MS^4(2) in Figure 1(a).

E. LODES of $Hex_{10}GlcNAc_2$

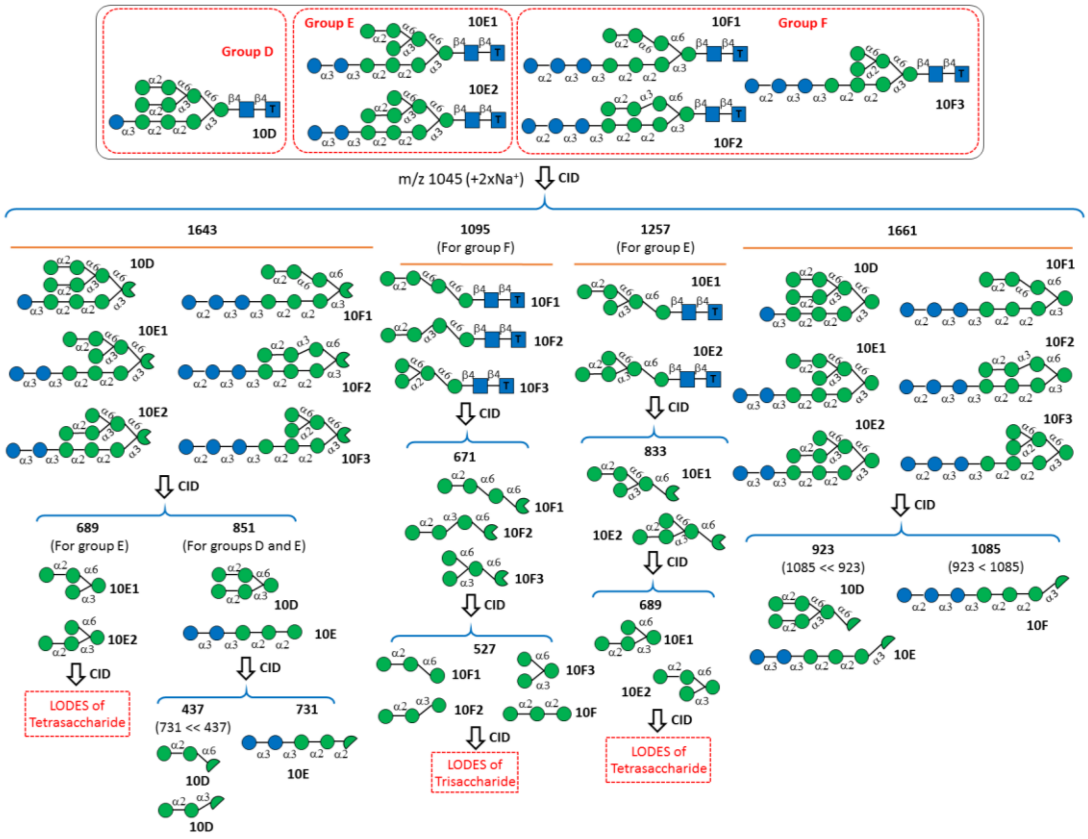

Supplementary Figure S6. LODES of $Hex_{10}GlcNAc_2$. The complete LODES of $Hex_{10}GlcNAc_2$.

F. Chromatograms of Eluents Collected From The Chromatogram in Figure 6(b)

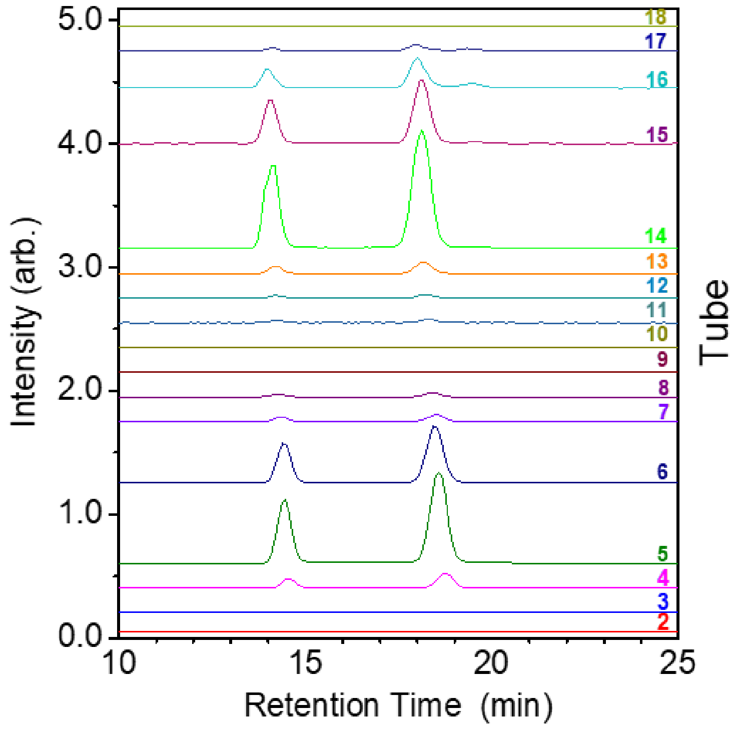

Supplementary Figure S7. Chromatograms of eluents collected from the chromatogram in Figure 6(b). The eluents corresponding to the chromatogram in Figure 6(b) were collected every 30 seconds. After repeating the collection of 10 times, the eluents were stored in collecting tubes at room temperature for 6 hours. Then the eluents were concentrated and reinjected into the same HPLC separately. Two peaks are located at the same retention of Figure 6(b), the relative intensities of these two peaks in each chromatogram are the same as that of Figure 6(b), and tubes 5 and 14 (corresponding to the retention time 13.5-14 and 18.0-18.5 minutes in chromatogram of Figure 6 (b)) have large intensity. They indicate two peaks in Figure 6(b) belong to one isomer and they are only different by the anomericity at the reducing end.

G. NMR Spectra and Assignments of Synthesized N-glycans

(1) 5-Aminopentyl-5-acetamido-3,5-dideoxy-D-glycero-β-D-galacto-non-2-ulopyranosylate]-(2→3)-β-D-galactopyranosyl-(1→4)-2-acetamido-2-deoxy-β-D-glucopyranosyl) -(1→2) 2-acetamido-2-deoxy-β-D-glucopyranosyl)-(1→6),(-α D-mannopyranosyl]-(1→6), 2-acetamido-2-deoxy-β-D-glucopyranosyl-(1→2)-α-D-mannopyranosyl]-(1→3)-β-D-mannopyranosyl-(1→4)-2-acetamido-2-deoxy-β-D-glucopyranosyl-(1→4)-2-acetamido-2-deoxy-β-D-glucopyranoside (12).

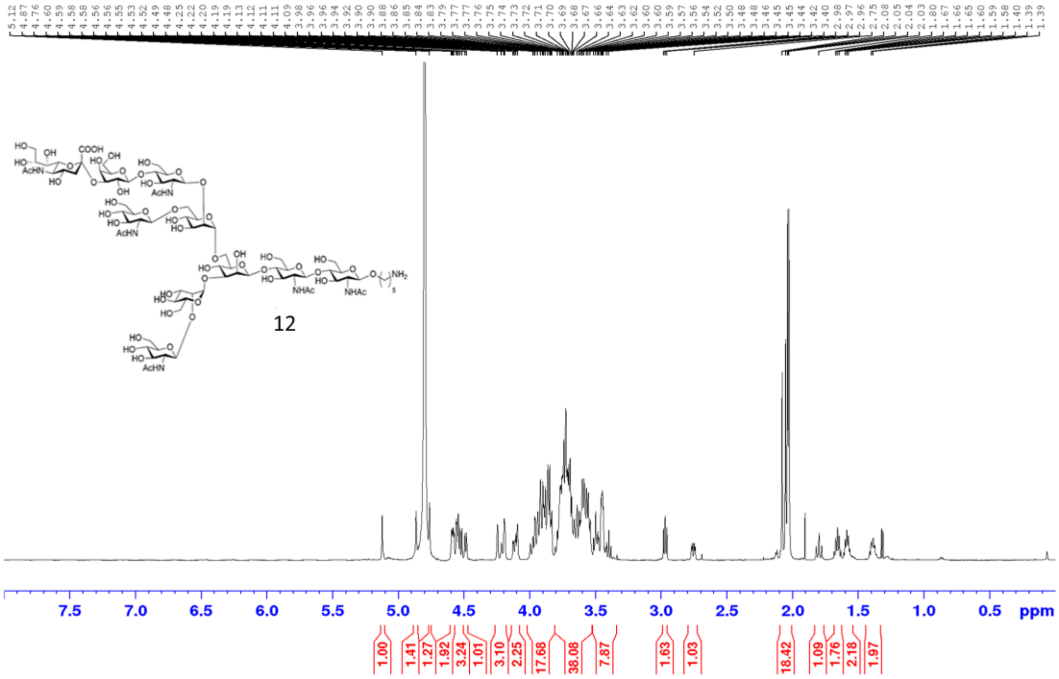

Figure S8. ^{1}**H NMR (600 MHz, Deuterium Oxide)** δ 5.12 (d, J = 1.6 Hz, 1H), 4.87 (s, 1H), 4.77 (s, 1H), 4.91 – 4.85 (m, 1H), 4.92 – 4.85 (m, 1H), 4.60 (m, 2H), 4.58 – 4.51 (m, 3H), 4.51 – 4.40 (m, 2H), 4.27 – 4.16 (m, 4H), 4.12 – 4.07 (m, 1H), 4.01 – 3.80 (m, 20H), 3.80 – 3.52 (m, 39H), 3.52 – 3.35 (m, 9H), 3.00 – 2.91 (m, 2H), 2.71 – 2.64 (m, 1H), 2.08 (d, J = 2.2 Hz, 4H), 2.07 (s, 3H), 2.06 (s, 6H), 2.05 – 2.04 (m, 6H), 2.03 (s, 3H), 2.03 (s, 3H), 1.72 (t, J = 12.2 Hz, 1H), 1.68 – 1.62 (m, 2H), 1.59 (p, J = 6.6 Hz, 2H), 1.38 (dtd, J = 15.1, 7.2, 4.2 Hz, 2H).

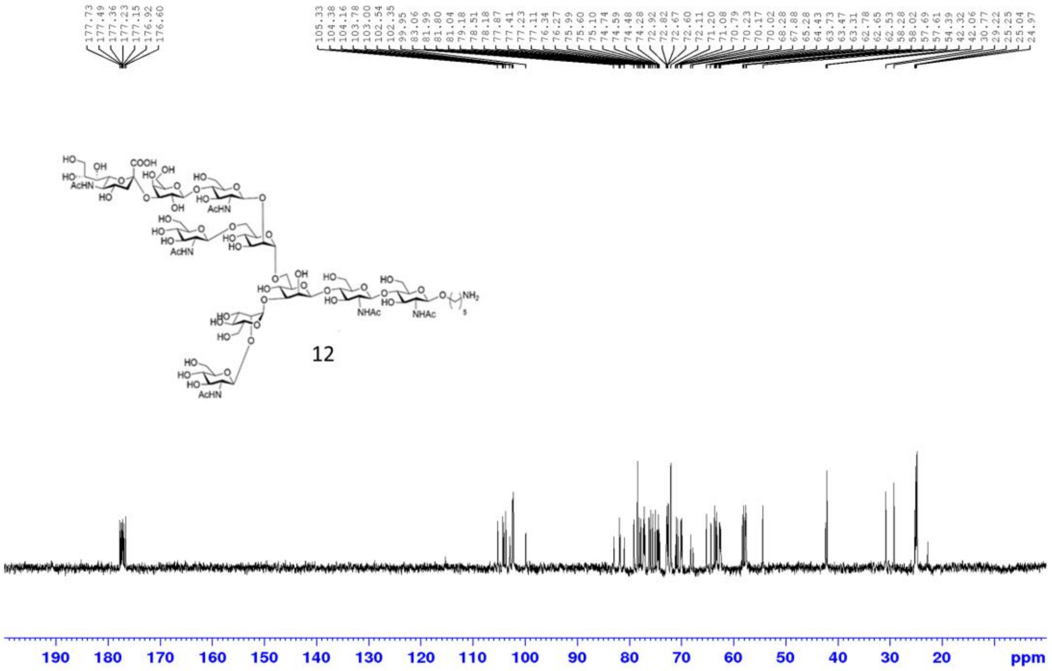

Figure S9. ^{13}**C NMR (151 MHz,** D_2O**)** δ 177.6, 177.5, 177.4, 177.3, 177.2, 177.0, 176.9, 176.3, 173.8, 106.3, 105.7, 104.4, 104.3, 104.1, 103.8, 103.2, 102.9, 102.4, 102.3, 102.2, 99.9, 83.3, 83.1, 82.2, 82.0, 79.2, 78.5, 78.1, 77.4, 77.2, 77.2, 77.1, 76.4, 76.3, 76.0, 75.3, 75.2, 75.1, 74.8, 74.7, 74.6, 74.4, 74.3, 73.7, 73.5, 73.0, 72.8, 72.7, 72.6, 72.1, 72.1, 71.2, 71.1, 71.1, 70.9, 70.2, 70.0, 68.4, 68.3, 66.0, 65.4, 64.4, 63.7, 63.5, 63.3, 63.0, 62.8, 62.6, 62.5, 58.3, 58.0, 57.7, 57.6, 57.4, 54.6, 42.8, 42.1, 30.8, 29.4, 29.4, 26.0, 25.3, 25.2, 25.1, 25.0, 25.0, 25.0, 24.8, 24.8, 22.0.

(2) 5-Aminopentyl-5-acetamido-3,5-dideoxy-D-glycero-β-D-galacto-non-2-ulopyranosylate]-(2→6)-β-D-galactopyranosyl-(1→4)-2-acetamido-2-deoxy-β-D-glucopyranosyl) -(1→6) 2-acetamido-2-deoxy-β-D-glucopyranosyl)-(1→2),(-α D-mannopyranosyl]-(1→6), 2-acetamido-2-deoxy-β-D-glucopyranosyl-(1→2)-α-D-mannopyranosyl]-(1→3)-β-D-mannopyranosyl-(1→4)-2-acetamido-2-deoxy-β-D-glucopyranosyl-(1→4)-2-acetamido-2-deoxy-β-D-glucopyranoside (25).

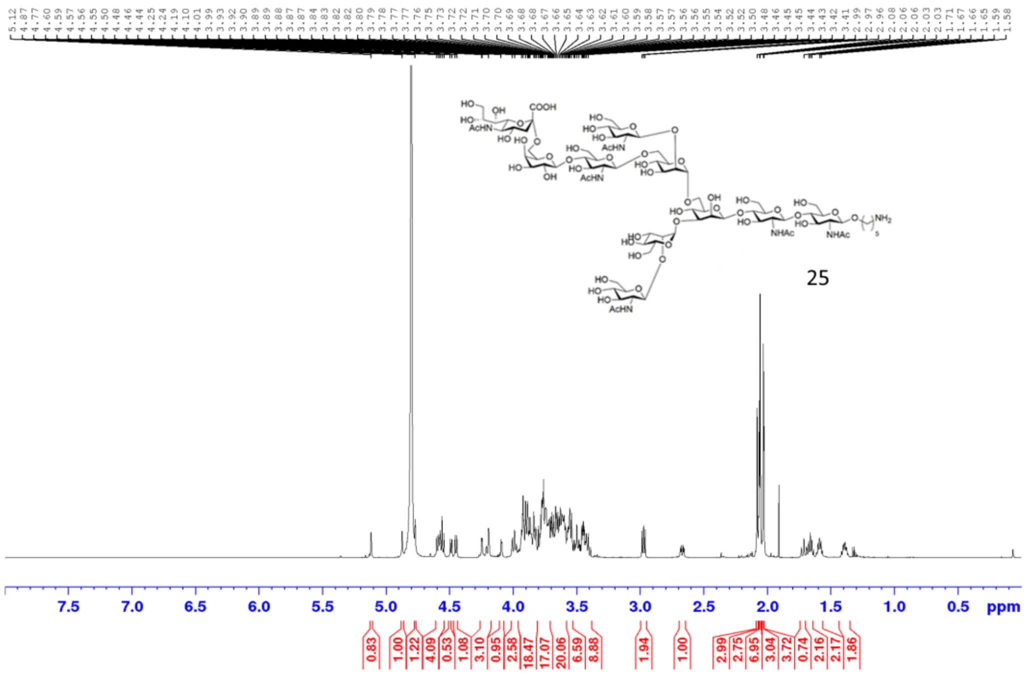

Figure S10. ^{1}**H NMR (600 MHz, Deuterium Oxide)** δ 5.13 (s, 1H), 4.86 (s, 1H), 4.76 (s, 1H), 4.59 (d, *J* = 7.6 Hz, 1H), 4.56 (m, 4H), 4.49 (m, 1H), 4.25 (d, *J* = 3.2 Hz, 1H), 4.22 – 4.17 (m, 2H), 4.15 – 4.07 (m, 2H), 4.01 (dd, *J* = 12.3, 2.2 Hz, 1H), 3.95 (dd, *J* = 11.8, 2.8 Hz, 2H), 3.90 – 3.89 (m, 5H), 3.89 – 3.80 (m, 11H), 3.80 – 3.67 (m, 24 H), 3.67 – 3.52 (m, 19 H), 3.52 – 3.38 (m, 9 H), , 2.96 (dd, *J* = 8.5, 6.8 Hz, 2H), 2.76 (dd, *J* = 12.4, 4.6 Hz, 1H), 2.07 (s, 1H), 2.05 (s, 3H), 2.04 (s, 3H), 2.07 – 2.01 (m, 12H), 1.80 (t, *J* = 12.2 Hz, 1H), 1.65 (q, *J* = 7.7 Hz, 2H), 1.62 – 1.55 (m, 2H), 1.44 – 1.33 (m, 2H).

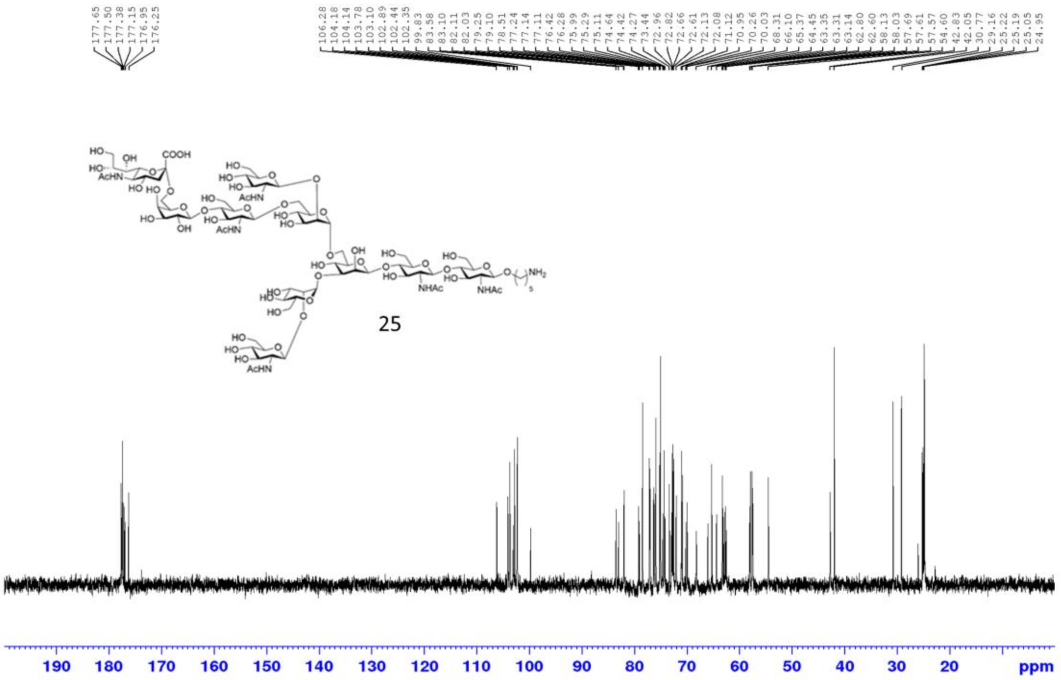

Figure S11. **^{13}C NMR (151 MHz, D_2O)** δ 177.7, 177.5, 177.4, 177.3, 177.2, 176.9, 176.6, 105.3, 104.4, 104.1, 103.8, 103.1, 102.6, 102.4,102.3, 99.9, 83.0, 82.1, 82.0, 81.0, 79.2, 79.2, 78.5, 78.2, 77.9, 77.4, 77.2, 77.1, 76.3, 76.0, 75.6, 75.1, 75.0, 74.7, 74.6, 74.5, 74.3, 73.0, 72.9, 72.8, 72.7, 72.6, 72.1, 71.1, 70.8, 70.3, 70.2, 70.0, 68.4, 68.2, 65.3, 64.5, 63.8, 63.3, 62.8, 62.6, 58.1, 58.0, 57.8, 57.7, 57.6, 54.4, 42.3, 42.1, 30.8, 29.3, 26.0, 25.2, 25.1, 24.9, 24.8, 24.8.

(3) 5-Aminopentyl-5-acetamido-3,5-dideoxy-D-glycero-β-D-galacto-non-2-ulopyranosylate]-(2→3)-β-D-galactopyranosyl-(1→4)-2-acetamido-2-deoxy-β-D-glucopyranosyl) -(1→2), (-α D-mannopyranosyl]-(1→6), -[bis-(2-acetamido-2-deoxy-β-D-glucopyranosyl)-(1→2), (1→4) -α-D-mannopyranosyl]-(1→3)-β-D-mannopyranosyl-(1→4)-2-acetamido-2-deoxy-β-D-glucopyranosyl-(1→4)-2-acetamido-2-deoxy-β-D-glucopyranoside. (13).

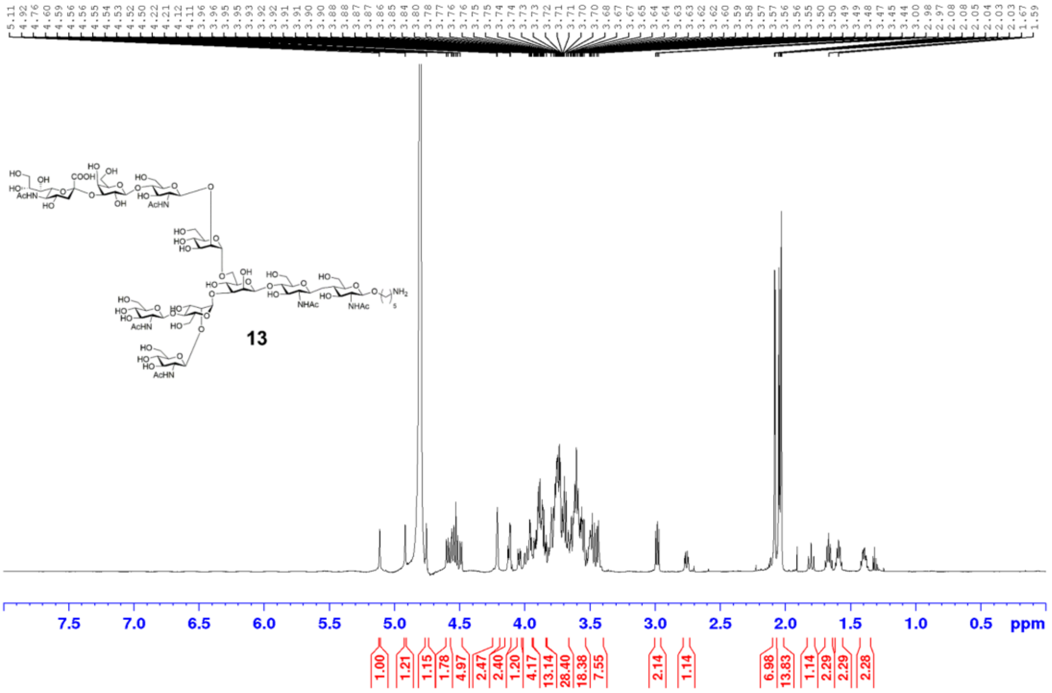

Figure S12. ^{1}H NMR (600 MHz, Deuterium Oxide) δ 5.12 (d, *J* = 1.8 Hz, 1H), 4.94 (d, *J* = 1.7 Hz, 1H), 4.76 (s, 1H), 4.60 (m, 2H), 4.53 (m, 2H), 4.49 (d, *J* = 7.6 Hz, 1H), 4.46 (m, 1H), 4.21 (dd, *J* = 3.3, 1.9 Hz, 2H), 4.11 (dd, *J* = 3.5, 1.6 Hz, 1H), 4.04 (dd, *J* = 9.3, 3.3 Hz, 1H), 4.02 – 3.83 (m, 19H), 3.83 – 3.71 (m, 21H), 3.71 – 3.52 (m, 31H), 3.52 – 3.39 (m, 8H), 3.01 – 2.95 (m, 2H), 2.67 (dd, *J* = 12.4, 4.7 Hz, 1H), 2.08 (s, 3H), 2.08 (s, 3H), 2.06 (s, 3H), 2.05 (s, 3H), 2.03 (s, 3H), 2.03 (s, 3H), 2.03 (s, 3H), 1.76 – 1.69 (m, 1H), 1.66 (q, *J* = 7.8 Hz, 2H), 1.59 (q, *J* = 6.6 Hz, 2H), 1.39 (ddt, *J* = 15.2, 7.3, 4.5 Hz, 2H).

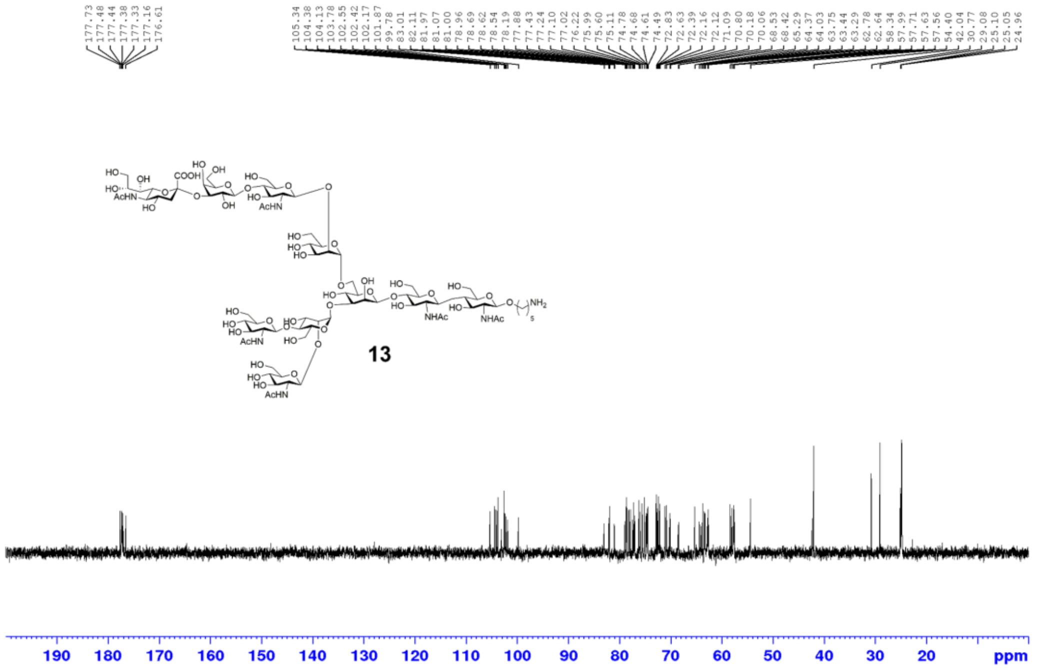

Figure S13. ^{13}C NMR (151 MHz, D_2O) δ 177.7, 177.5, 177.4, 177.3, 177.2, 176.3, 106.3, 104.4, 104.1, 103.8, 103.2, 102.9, 102.4, 102.0, 101.9, 99.7, 83.4, 83.0, 82.3, 82.0, 81.1, 78.9, 78.7, 78.6, 78.5, 77.2, 77.1, 77.1, 77.0, 76.4, 76.2, 76.0, 75.6, 75.3, 75.1, 74.9, 74.7, 74.6, 74.4, 73.5, 72.9, 72.8, 72.6, 72.4, 72.2, 71.1, 71.1, 70.9, 70.8, 70.0, 68.6, 68.5, 66.0, 65.4, 64.3, 64.0, 63.4, 63.3, 63.0, 62.8, 62.7, 58.3, 58.0, 57.7, 57.6, 57.4, 54.6, 42.8, 42.0, 30.8, 29.1, 25.1, 25.0, 24.9, 24.8, 24.8.

(4) 5-Aminopentyl-5-acetamido-3,5-dideoxy-D-glycero-β-D-galacto-non-2-ulopyranosylate]-(2→3)-β-D-galactopyranosyl-(1→4)-2-acetamido-2-deoxy-β-D-glucopyranosyl) - (1→2) - 2-acetamido-2-deoxy-β-D-glucopyranosyl-(1→4)-α-D-mannopyranosyl]-(1→3), [bis-(2-acetamido-2-deoxy-β-D-glucopyranosyl)-(1→2), (1→6)-α-D-mannopyranosyl]-(1→6)-β-D-mannopyranosyl-(1→4)-2-acetamido-2-deoxy-β-D-glucopyranosyl-(1→4)-2-acetamido-2-deoxy-β-D-glucopyranoside (17).

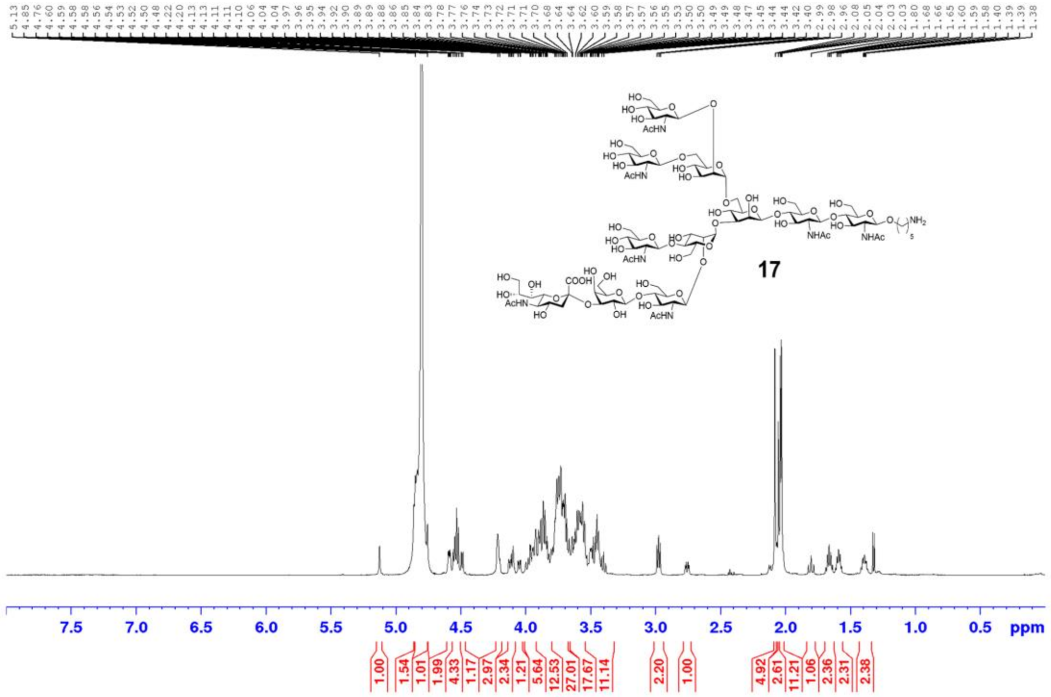

Figure S14. ¹H NMR (600 MHz, Deuterium Oxide) δ 5.13 (s, 1H), 4.86 (s, 1H), 4.76 (s, 1H), 4.60 – 4.43 (m, 7H), 4.23 – 4.17 (m, 3H), 4.11 – 4.02 (m, 2H), 4.02 – 3.97 (m, 2H), 3.96 – 3.85 (m, 11H), 3.85 – 3.79 (m, 7H), 3.76 (d, *J* = 7.1 Hz, 4H), 3.79 – 3.68 (m, 18H), 3.67 – 3.63 (m, 6H), 3.63 – 3.51 (m, 14H), 3.50 – 3.38 (m, 9H), 2.98 (t, *J* = 7.7 Hz, 1H), 2.66 (dd, *J* = 12.4, 4.7 Hz, 1H), 2.07 (s, 3H), 2.07 (s, 3H), 2.07 (s, 3H), 2.04 (s, 3H), 2.04 (s, 3H), 2.02 (s, 3H), 2.02 (s, 3H), 2.02 (s, 3H), 1.72 (t, *J* = 12.1 Hz, 1H), 1.68 – 1.55 (m, 4H), 1.39 (td, *J* = 7.8, 2.9 Hz, 2H).

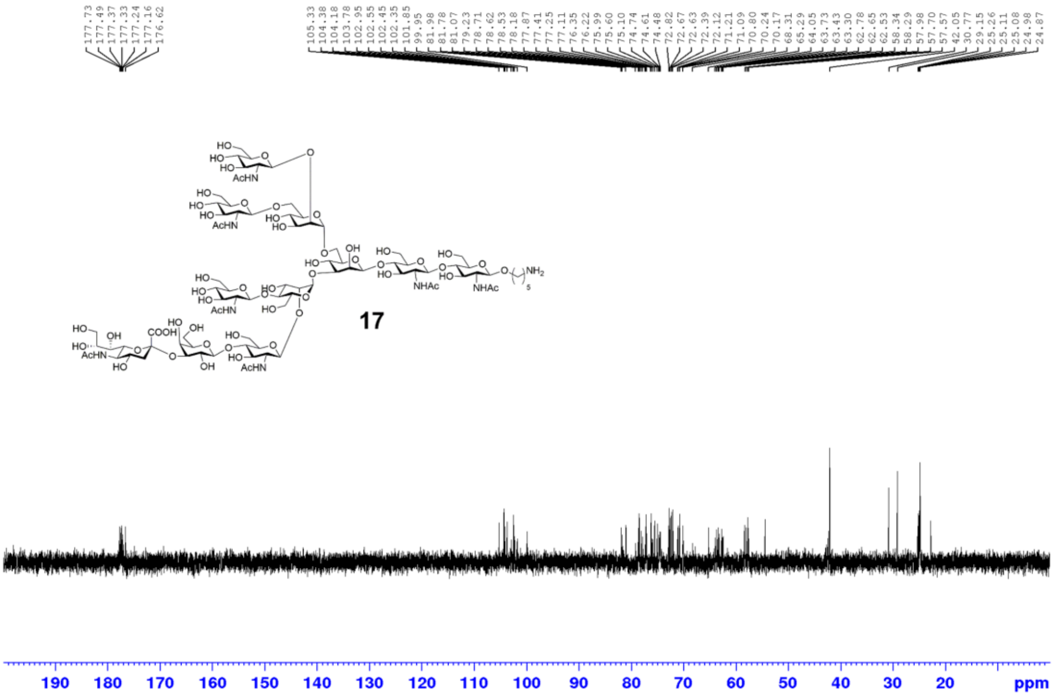

Figure S15. ^{13}C NMR (126 MHz, D_2O) δ 177.6, 177.3, 177.1, 177.0, 176.3, 106.3, 104.4, 104.1, 103.8, 103.1, 102.9, 102.4, 102.1, 101.9, 99.9, 83.5, 83.1, 82.0, 81.1, 79.2, 78.7, 78.6, 78.5, 77.2, 77.1, 76.4, 76.2, 76.0, 75.2, 75.1, 74.8, 74.6, 74.4, 74.3, 73.4, 72.9, 72.8, 72.7, 72.4, 72.1, 71.1, 70.9, 70.3, 68.4, 66.1, 65.4, 64.0, 63.5, 63.3, 63.1, 62.8, 62.6, 58.3, 58.1, 57.7, 57.6, 57.3, 54.6, 42.8, 42.0, 32.3, 30.8, 29.1, 25.2, 25.0, 24.9, 24.8, 24.8, 24.7.

(5) 5-Aminopentyl-5-acetamido-3,5-dideoxy-D-glycero-β-D-galacto-non-2-ulopyranosylate]-(2→6)-β-D-galactopyranosyl-(1→4)-2-acetamido-2-deoxy-β-D-glucopyranosyl)-(1→4)-2-acetamido-2-deoxy-β-D-glucopyranosyl-(1→2)-α-D-mannopyranosyl]-(1→3), [bis-(2-acetamido-2-deoxy-β-D-glucopyranosyl)-(1→2), (1→6)-α-D-mannopyranosyl]-(1→6)-β-D-mannopyranosyl-(1→4)-2-acetamido-2-deoxy-β-D-glucopyranosyl-(1→4)-2-acetamido-2-deoxy-β-D-glucopyranoside (28).

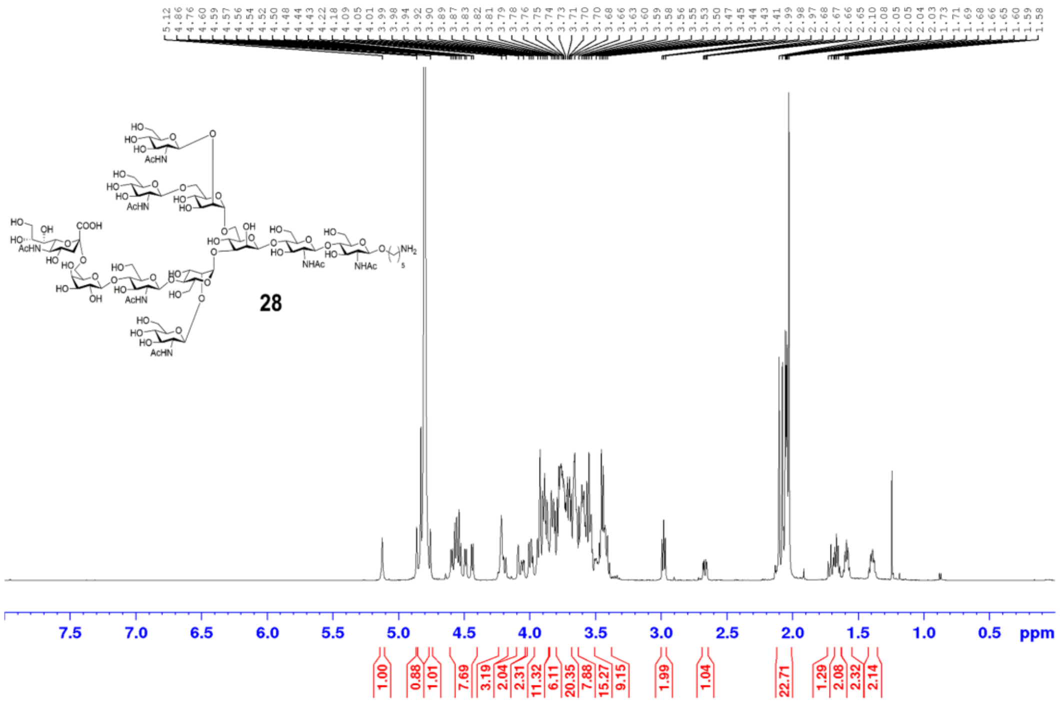

Figure S16. ^{1}H NMR (500 MHz, Deuterium Oxide) δ 5.11 (s, 1H), 4.85 (s, 1H), 4.75 (s, 1H), 4.61 – 4.46 (m, 7H), 4.21 (m, 3H), 4.14 – 4.07 (m, 2H), 4.03-3.98 (m, 2H), 3.97-3.93 (m, 2H), 3.93 – 3.82 (m, 15H), 3.82 – 3.70 (m, 29H), 3.67 – 3.51 (m, 22H), 3.51 – 3.39 (m, 10H), 2.96 – 2.93 (m, 2H), 2.76 (dd, J = 12.5, 4.6 Hz, 1H), 2.07 (s, 3H), 2.06 (s, 3H), 2.04 (s, 3H), 2.04 (s, 3H), 2.03 (s, 3H), 2.02 (s, 3H), 2.02 (s, 3H), 1.79 (t, J = 12.2 Hz, 1H), 1.63 (q, J = 7.7 Hz, 2H), 1.61 (dt, J = 29.3, 7.6 Hz, 2H), 1.39 (d, J = 7.9 Hz, 2H).

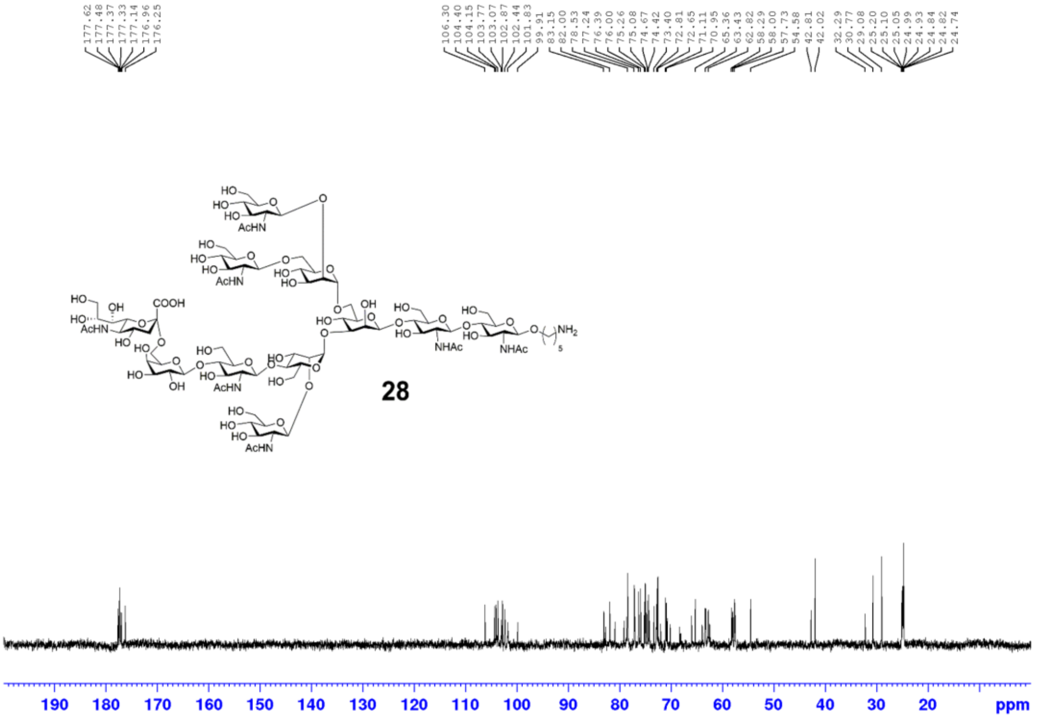

Figure S17. ^{13}**C NMR (126 MHz, D_2O)** δ 177.7, 177.5, 177.3, 177.3, 177.2, 177.0, 176.6, 164.3, 105.3, 104.4, 104.3, 104.1, 103.8, 103.1, 102.5, 102.5, 101.9, 99.9, 82.0, 81.0, 80.7, 79.2, 78.8, 78.5, 78.1, 77.9, 77.5, 77.2, 77.1, 76.4, 76.0, 75.6, 75.1, 74.9, 74.7, 74.5, 74.3, 73.7, 72.9, 72.7, 72.1, 71.1, 70.8, 70.2, 65.3, 64.0, 63.8, 63.4, 62.8, 62.6, 58.3, 58.1, 58.0, 57.9, 57.7, 57.6, 54.4, 42.3, 42.1, 30.8, 29.5, 25.2, 25.1, 25.1, 24.9, 24.9, 24.8, 24.7.
